# Platelet enzymatic activities in patients with late-onset schizophrenia spectrum disorders

**DOI:** 10.1192/j.eurpsy.2024.1334

**Published:** 2024-08-27

**Authors:** T. Prokhorova, I. Boksha, O. Savushkina, E. Tereshkina, E. Vorobyeva, G. Burbaeva

**Affiliations:** FSBSI “MENTAL HEALTH RESEARCH CENTRE”, Moscow, Russian Federation

## Abstract

**Introduction:**

Impairments in energy metabolism, glutamate neurotransmitter and antioxidant systems contribute substantially in development of schizophrenia spectrum disorders, especially in late-onset psychosis (LOP).

**Objectives:**

Revealing subgroups of patients with LOP by determining activity of platelet enzymes of energy, glutamate, and glutathione metabolism.

**Methods:**

62 women of 52-89 years old were studied, with late onset schizophrenia spectrum disorders (F20.0, F25, F22.0, F06.2 by ICD-10). PANSS with its subscales was used to assess the severity of psychotic symptoms. Scores by PANSS and activity levels of platelet cytochrome *c*-oxidase (COX), glutamate dehydrogenase (GDH), glutathione reductase (GR) and glutathione-S-transferase (GST) were evaluated twice: before and on the 28-th day of antipsychotic treatment. Activities of COX, GDH, GR, and GST were measured in 37 women of 50-84 years old comprising the control group.

**Results:**

Clustering of patients by the enzymatic activities resulted in 2 clusters (C1 and C2) significantly different by COX and GST (p<0.001). In C1 (n=40), as compared with control, reduced level of GDH activity before and after treatment (p=0.049 and p=0.032, respectively) and a reduced level of GR activity before treatment (p=0.026) were revealed. In C2 (n=22), as compared with the control, COX activity was increased before and after treatment (p=0.0001), GDH activity was decreased before and after treatment (p=0.0002 and p=0.0001, respectively), and GST activity was decreased before and after treatment (p=0.029 and p=0.0029, respectively). GR activity was not significantly changed in both clusters. Significant correlations were found between enzymatic activities and scores by psychometric scales: in C1, GR activity positively correlated with the score reduction (delta) by PANSS-Pos (R=0.45, p=0.004), by PANSS-Psy (R=0.44, p=0.005), and by PANSS (R=0.47, p=0.002), and GST activity – with the score reduction by PANSS-Psy (R=0.315, p=0.048). In C2 (n=22), GDH activity negatively correlated with the score reduction by PANSS-Pos (R=-0.41, p=0.050) and by PANSS (R=-0.49, p=0.021).

**Conclusions:**

The different correlations revealed in two separated clusters between enzymatic activity levels and clinical measures characterizing the antipsychotic treatment efficacy will allow us to approach differentiated predicting the effectiveness of pharmacotherapy using the biochemical parameters.

**Disclosure of Interest:**

None Declared

